# Supplementary material for: Development of D-box peptides to inhibit the anaphase-promoting complex/cyclosome
Source: eLife. 2025 Sep 1;14:RP104238. doi: 10.7554/eLife.104238 (PMC12401543; doi:10.7554/eLife.104238)
Supplement: Figure 6—figure supplement 1—source data 4. [file elife-104238-fig6-figsupp1-data4.zip › Figure_S4_source_data_4/20180826_20m_exposure.pdf]

20180826 CETSA dataset

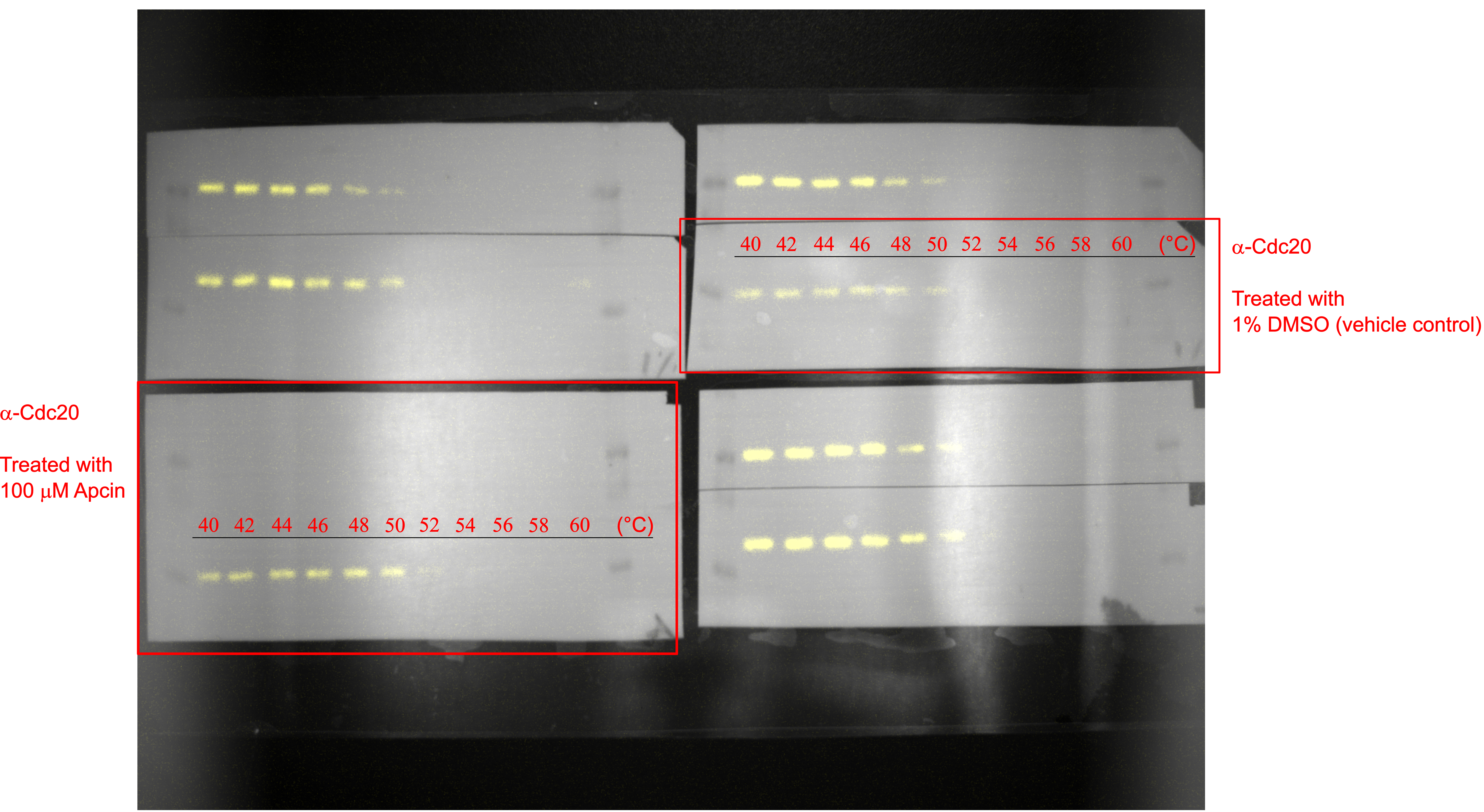

Figure 6-figure supplement 5, Source data 4. Red box highlights data that are used and plotted in Figure 6-figure supplement 5. Source data 4 relates to one experimental replicate (collected on 20180826) performing a CETSA experiment detecting endogenous Cdc20 in a HEK293T cell lysate. Temperatures indicated correspond to that of which the lysate aliquot was heated during the CETSA experiment, prior to western blot detection.
