# Supplementary material for: Development of D-box peptides to inhibit the anaphase-promoting complex/cyclosome
Source: eLife. 2025 Sep 1;14:RP104238. doi: 10.7554/eLife.104238 (PMC12401543; doi:10.7554/eLife.104238)
Supplement: Supplementary file 1. [file elife-104238-supp1.docx]

**Table A.** Data collection, phasing and refinement statistics for Cdc20 crystal structures with D-box peptides

| Crystal structure | Cdc20 – D21 | Cdc20 – D20 | Cdc20 – D7 |
| --- | --- | --- | --- |
| PDB accession code | 9I68 | 9I69 | 9I6A |
| **Data collection** |  |  |  |
| Space group | P2_1_ | P2_1_ | P2_1_ |
| Unit cell, a, b, c (Å), α, β, γ (°) | 35.49, 87.55, 48.57 90.00, 109.71, 90.00 | 35.34, 87.34, 48.51 90.00, 110.14, 90.00 | 35.00, 86.87, 48.03 90.00, 109.60, 90.00 |
| Resolution range, Å | 45.73 - 1.51 (1.66 - 1.51) | 45.55 - 1.46 (1.60 - 1.46) | 45.24 - 1.92 (2.09 - 1.92) |
| Total reflections | 162715 (6076) | 184416 (10465) | 76640 (3657) |
| Unique reflections | 32820 (1641) | 35450 (1772) | 14979 (750) |
| Multiplicity | 5.0 (3.7) | 5.2 (5.9) | 5.1 (4.9) |
| Completeness (spherical), % | 74.7 (14.5) | 74.5 (16.0) | 71.8 (15.4) |
| Completeness (ellipsoidal), % | 90.4 (41.9) | 92.9 (60.1) | 90.5 (57.6) |
| I/σI | 13.1 (1.6) | 13.6 (1.4) | 7.6 (1.5) |
| R_merge_ | 0.050 (0.598) | 0.048 (1.000) | 0.132 (1.161) |
| CC_1/2­_ | 0.999 (0.714) | 0.999 (0.635) | 0.996 (0.536) |
| **Refinement** |  |  |  |
| R_work_/R_free_, % | 0.175/0.194 | 0.165/0.184 | 0.207/0.231 |
| Unique reflections used | 32820 | 35450 | 14966 |
| rmsd bond lengths, Å | 0.008 | 0.008 | 0.008 |
| rmsd bond angles, ° | 1.01 | 1.02 | 1.00 |
| Ramachandran analysis: |  |  |  |
| Favoured, % | 97.10 | 98.06 | 97.33 |
| Allowed, % | 2.58 | 1.61 | 2.33 |
| Outliers, % | 0.32 | 0.32 | 0.33 |
| Number of atoms  (average B-factor, Å^2^) |  |  |  |
| Protein | 2452 (24.31) | 2434 (25.80) | 2340 (29.68) |
| Peptide | 46 (37.72) | 46 (46.88) | 25 (48.49) |
| Solvent | 236 (39.17) | 240 (44.08) | 72 (32.96) |
| Mean/Wilson B-factor, Å^2^ | 25.8/22.9 | 27.8/23.9 | 30.0/27.8 |
